# Understanding Mind-Body Interaction from the Perspective of East Asian Medicine

**DOI:** 10.1155/2017/7618419

**Published:** 2017-08-22

**Authors:** Ye-Seul Lee, Yeonhee Ryu, Won-Mo Jung, Jungjoo Kim, Taehyung Lee, Younbyoung Chae

**Affiliations:** ^1^Acupuncture and Meridian Science Research Center, College of Korean Medicine, Kyung Hee University, Seoul, Republic of Korea; ^2^KM Fundamental Research Division, Korea Institute of Oriental Medicine, Daejeon, Republic of Korea

## Abstract

**Objective:**

Attempts to understand the emotion have evolved from the perspective of an independent cognitive system of the mind to that of an interactive response involving the body. This study aimed to quantify and visualize relationships between different emotions and bodily organ systems from the perspective of East Asian medicine.

**Methods:**

Term frequency-inverse document frequency (tf-idf) method was used to quantify the significance of Five Viscera and the gallbladder relative to seven different emotions through the classical medical text of DongUiBoGam. Bodily organs that corresponded to different emotions were visualized using a body template.

**Results:**

The emotions had superior tf-idf values with the following bodily organs: anger with the liver, happiness with the heart, thoughtfulness with the heart and spleen, sadness with the heart and lungs, fear with the kidneys and the heart, surprise with the heart and the gallbladder, and anxiety with the heart and the lungs. Specific patterns between the emotions and corresponding bodily organ systems were identified.

**Conclusion:**

The present findings will further the current understanding of the relationship between the mind and body from the perspective of East Asian medicine. Western medicine characterizes emotional disorders using “neural” language while East Asian medicine uses “somatic” language.

## 1. Introduction

Throughout the history of science, efforts and discussion aimed at comprehending emotions in relation to the body have focused on the manifestation of emotions through physiological responses [1–4]. Subsequent to James and Lange proposing that physiological feedback plays a role in emotion [5, 6], more recent findings have indicated that there are close relationships between emotions and bodily responses during the formation of mental experiences. This process, which may also be referred to in terms of feelings, provides evidence that physiological reactions such as the activation of the cardiovascular, skeletomuscular, neuroendocrine, and autonomic nervous systems and their somatosensory feedback mechanisms trigger emotional experiences [4, 7, 8]. Furthermore, it has been demonstrated that specific patterns of bodily sensations are related to categorical emotions [9]. A recent perspective on the relationships between emotion and bodily responses indicates that there are direct and instantaneous interactions within the body that allow for the experience of an emotion to be defined as a mental recognition as well as a feeling within the body.

The intangible nature of emotion has been a subject of interest since the beginning of recorded history. Although scholars from diverse fields such as medicine, neuroscience, and anthropology have taken many different approaches to address this issue, the relationship between emotion (psychological processes) and the body (somatic system) has been a primary focus. The thoughts and language of modern Western scholars are strongly influenced by the dichotomy of the psyche and the soma, which implies the superiority of the intentional mind over the body [10]. On the other hand, East Asian perspectives understand emotions and emotional disorders through the body and associate emotions with different parts of the bodily system, such as the liver or heart [11–13]. Moreover, East Asian medicine interprets the human body and its disorders, including emotional and psychosomatic disorders, using a holistic approach [14]. In other words, East Asian medicine understands emotions in terms of their relationships with different visceral systems in the body [15]. Although imaginary in nature, metaphors involving internal organs that evoke imaginary bodily images are not really arbitrary but appear to have bodily and/or psychological bases [16]. This approach enables one to understand the mind and body within a unified framework in which different functions and components of the bodily system are related to corresponding categorical emotions.

In East Asian medicine, visceral systems do not exactly correspond to anatomical organs as described in modern human anatomy but, rather, reflect functional systems that encompass many different functional realms [17]. The close relationships among these systems allow for the direct association of emotions with specific physical symptoms within certain visceral systems as well as an understanding of emotional regulation via the visceral system, as perceived in East Asian medicine by the movement of *qi* [18]. Two different exemplar sentences from* Huangdi Neijing*, which has been the fundamental doctrinal source of East Asian medicine for two millennia, illustrate a singular relationship between emotion and the visceral system: “the liver is in charge of anger” and “anger damages liver.” These sentences imply a bidirectional and instantaneous association between an emotion and the body under normal and pathogenic conditions of health. The* Huangdi Neijing* further describes the manifestation of this relationship in the body as it is mediated by *qi* movement such that “anger (is) the *qi* movement surging upwards.” From the perspective of East Asian medicine, the liver is involved in sending *qi* upwards during its circulation, and excerpts from this classical medical text elaborately describe the manner in which the liver, anger, and the ascending movement of *qi* are different phenomena that are part of a single systemic function within the body [15]. Although it is invisible, *qi* is felt throughout the body via sensations such as heat and cold or numbness and pain. Furthermore, according to East Asian medicine, because the movement of *qi* is governed by the visceral systems of the liver, heart, spleen, lungs, and kidneys, it can be defined as a part of the body system that operates through different processes and that is recognized through bodily sensations [19]. While recent scientific research has indicated that specific bodily sensations are related to specific emotions, East Asian medicine has demonstrated that certain movements of *qi* can be recognized as specific categorical emotions [9, 15]. Additionally, East Asian medicine relates to emotions through the Five Viscera rather than the brain [20].

Thus, the present study examined the relationship between emotion and the body from the perspective of East Asian medicine using a data mining process in a representative classical medical text. This study aimed to describe how the relationship between emotion and the body is understood as a whole by quantifying and visualizing these relationships in the classical medical text of* DongUiBoGam.*

## 2. Methods

### 2.1. Data Source

The classical medical text known as* DongUiBoGam* was first published in 1613 by a royal physician, Heo Jun (1539–1615), under the Joseon Dynasty and is widely considered to be the representative doctrine of Korean medicine [21]. This text serves as the fundamental doctrinal source of medical classics such as the* Huangdi Neijing *and* ShangHan Lun* as well as more recent medical texts, including the* Wanbinghuichun *(1587) from China and the* HyangYakJipSeongBang *(1443) and* EuiRimChwalYo* (1567) from Korea. In fact, the* DongUiBoGam* was written as the summation of East Asian medicine knowledge up to the year of its compilation and served as the core text for medical theories and perspectives as understood in Korean medicine [22].

The essence of health as depicted in the* DongUiBoGam* emphasizes the harmony of the Five Viscera and the circulation of *qi* throughout the body. The picture of ShinHyungJangBuDo on the first page of the* DongUiBoGam* illustrates this concept by emphasizing the body organs, or viscera, and the major pathway through which the *qi* passes by breathing [22]. Thus, in the* DongUiBoGam*, emotions are categorized into seven different groups: anger, happiness, thoughtfulness, sadness, anxiety, fear, and surprise. Furthermore, each of these categorical emotions is described in terms of its close relationship to the visceral system and the flow of *qi*.

### 2.2. Database Construction

We included three chapters of text in the* DongUiBoGam*: the first chapter, NaeGyeong (internal bodily elements), addresses internal body elements such as* essence, qi, spirit*, and the Five Viscera and Six Bowels; the second chapter, Waehyung (external bodily elements), discusses disorders and symptoms that manifest in external body parts; and the last chapter, Japbyung (miscellaneous disorders), includes descriptions of miscellaneous diseases and details the causes of these symptoms or disorders. Different parts of the chapters and subchapters elaborately discuss categorical emotions and some passages illustrate the manner in which emotions can affect the bodily state and result in different symptoms that might also cause various health-related issues. Other passages describe how the visceral system regulates emotions and shows how sicknesses in these visceral systems can cause irregular emotional states or mental experiences within a patient.

Using these passages as the data source, information on emotions from the perspective of Korean medicine was collected as the dataset. Subsequently, mentions of the visceral system in these passages were collected and the data were categorized according to different emotions and different viscera. The collected data consisted of various groups of passages mentioning different categorical emotions (anger, happiness, thoughtfulness, sadness, anxiety, fear, and surprise) according to the visceral systems of the liver, heart, spleen, lung, kidneys, and gallbladder; notably, the gallbladder is the only organ system among the Six Bowels that is described in relation to emotions. The text of the* DongUiBoGam* explains the symptoms of disorders that are caused by either excessive or deficient activity within the gallbladder system through certain emotions, such as anger and surprise; as a result, the present study included the five visceral organs and the gallbladder in its analyses. After removing duplicate passages, a total of 334 sentences were analyzed in the present study.

### 2.3. Data Mining and Visualization

Using the above-described data construction, data regarding the cooccurrence of frequencies and visceral systems within emotional categories were extracted to understand relationships between these variables. In other words, the specific viscera that were more meaningfully associated with one category of emotion were determined by the application of a term frequency-inverse document frequency (tf-idf) weighting scheme to the cooccurrence table.

The tf-idf method is one of the most widely used weighting schemes in the data mining research field, especially for information retrieval systems, because it quantifies the significance of particular terms in a document [23]. Using this system, the present study quantified the significance of the associations between mentions of the body system and emotions. In the tf-idf scheme, term frequency (tf [*t* · *d*]) refers to the number of times that the term “*t*” occurs in document “*d*” and, therefore, tf_(*t*,*d*)_ represents how relevant term “*t*” is to document “*d*.” Document frequency (*d*_*ft*_) is the number of documents that contain the term “*t*” and, therefore, *d*_*ft*_ represents the rarity of a term within the system of documents. Across the document system, rare terms are more informative than frequent terms and, thus, the inverse document frequency of “*t*” (idf_*t*_) is positively related to the informativeness of “*t*.” Arithmetically, idf is defined as log⁡(*N*/*d*_*ft*_) instead of *N*/*d*_*ft*_ where *N* is the number of whole documents in order to diminish the effect of idf. Thus, in the present study, tf-idf_(*v*,*e*)_ was defined by assigning visceral organs as the “term” and emotions as the “document” so that the equation quantified the significance of the relationship between a specific visceral system and a specific emotion.

Based on the tf-idf_(*v*,*e*)_ values, each emotion was represented by a vector of the tf-idf weights in a 6-dimensional vector space. Next, the calculated tf-idf weights of each emotion were normalized using the cosine normalization. All data mining was done using the R package (https://r-project.org) and the relationship between an emotion and a particular visceral system was only described if it exhibited a tf-idf value > 0.2. The calculated values were overlaid on a human body template using matplotlib, which is a plotting library for python (http://matplotlib.org/); a variety of relationships between specific emotions and specific visceral systems were labeled according to the tf-idf_(*v*,*e*)_ values.

## 3. Results

### 3.1. Characteristics of the Relationships between Emotions and the Visceral Systems

**Figure 1:**
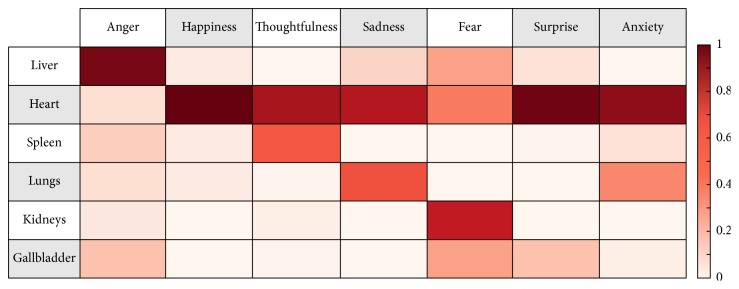
Characteristics of the relationships between emotions and the visceral systems. Emotions are presented on the *x*-axis of the array and the organs of the visceral system are presented on the *y*-axis of the array. The following emotions had superior tf-idf values with the following bodily organs: anger with the liver, happiness with the heart, thoughtfulness with the heart and spleen, sadness with the heart and lungs, fear with the kidneys and the heart, surprise with the heart and the gallbladder, and anxiety with the heart and the lungs.

The bodily organ systems, namely, the Five Viscera and the gallbladder, were highly associated with the seven emotions (Figure 1). More specifically, anger showed superior tf-idf values with the liver (0.94), happiness showed superior tf-idf values with the heart (0.99), thoughtfulness showed superior tf-idf values with the heart (0.84) and spleen (0.54), sadness showed superior tf-idf values with the heart (0.81) and lungs (0.56), fear showed superior tf-idf values with the kidneys (0.76), heart (0.45), liver (0.33), and gallbladder (0.33), surprise showed superior tf-idf values with the heart (0.97) and the gallbladder (0.23), and anxiety showed superior tf-idf values with the heart (0.90) and lungs (0.41).

### 3.2. Visualization of Specific Patterns between an Emotion and the Human Body

**Figure 2:**
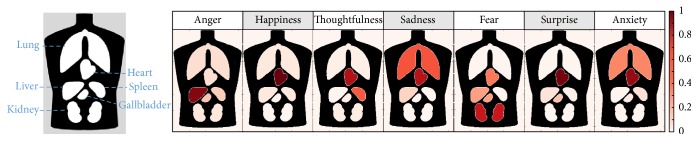
Visualization of specific patterns between an emotion and the human body. Seven categorical emotions were visualized on a human body template that included the lungs, heart, liver, gallbladder, spleen, and kidneys. Specific patterns existed between the visceral system and corresponding emotions.

The visualization of emotions on a human body template created for the present study (Figure 2) revealed that specific patterns existed between the visceral system and corresponding emotions such that anger corresponded with the liver, happiness with the heart, thoughtfulness with the heart and spleen, sadness with the heart and lungs, fear with the kidneys, heart, liver, and gallbladder, anxiety with the heart and lungs, and surprise with the heart and gallbladder. Furthermore, the present findings showed that the heart had significant associations with most of the emotions listed, other than anger and fear.

## 4. Discussion

The present study used a data mining procedure to analyze relationships between emotions and the visceral system according to the principles of East Asian medicine. Based on the normalized tf-idf values for the frequency of cooccurrences between the seven categorical emotions and six bodily organs, including the Five Viscera and the gallbladder, each of the categorical emotions was related to a specific bodily organ. Anger was related to the liver, happiness to the heart, thoughtfulness to the heart and spleen, sadness to the heart and lungs, fear to the kidneys, heart, liver, and gallbladder, surprise to the heart and the gallbladder, and anxiety to the heart and the lungs.

The present findings also demonstrated that specific patterns existed between the visceral system and corresponding emotions, which suggests that each emotion is primarily associated with a corresponding body system and can also be explained by the principles of East Asian medicine. Additionally, the patterns observed in the present study were similar to theories from East Asian medicine [15]. For example, the* DongUiBoGam* states the following: “Liver is in charge of anger, heart is in charge of happiness, spleen is in charge of thoughtfulness, lungs are in charge of sadness, and kidneys are in charge of fear.” The quantification of the terms used to explain the relationships between emotion and bodily organs in this classical medical text produced results that were similar to the principles of East Asian medicine. Furthermore, imbalances in emotions that can lead to illnesses in their corresponding organs can also be explained by the* DongUiBoGam*, which describes in detail the manner in which damage to these organ systems and the gallbladder are manifested through excessive or abnormal emotional states. For example, this text states the following: “Anger damages liver, happiness damages heart, thoughts damage spleen, anxiety damages lungs, fear damages kidneys, and surprise damages gallbladder.”

Thus, the present study provides evidence, from the perspective of East Asian medicine, that emotions are related to visceral organs, a finding that differs from the perspective of Western medicine in which emotions are understood in terms of their relationship with specific brain areas, such as the amygdala [24, 25]. The comprehensive role of the heart in East Asian medicine which includes the role of the brain in Western medicine implicates the somatization of the mind as illustrated by previous studies. By understanding the emotions in the context of the body itself rather than in a dichotomized model, the relationship of the mind and the body is horizontal [10]. These findings also showed that the heart was significantly prevalent in most emotions, which implies that this organ is considered to be a common visceral system involved in the experience of emotions. Interestingly, many other East Asian medical texts also consider the heart to be the center of the mind and emotions. For example, the sentences “[The] heart stores the mind” and “Sadness, thoughtfulness, and worries all damage the heart” signify the role of the heart in the processing of thoughts and emotions.

It is also important to note that Porkert pointed out that the visceral systems in East Asian medicine, such as the “liver,” “heart,” and “spleen,” do not refer to these specific anatomical substrates but, rather, to a certain pattern of functions within the body [26, 27]. The organ-based nosology used in East Asian medicine is a metaphor for which the primary referent is not a particular anatomic organ but an emotion, diagnosed based on the patterns of somatic symptoms. Thus, East Asian medicine is built on a symptom-based language rather than an organ-based language [10]. Dualistic thought limits the bodily perceptions as well as bodily awareness, which dichotomizes our selfhood from our body, that is, the mind thinking of the body rather than the body perceiving itself [10].

Although the direct relationship between emotions and the body is characterized differently by East Asian and Western medicine traditions, efforts to overcome the limits of the mind-body dichotomy have been continuously suggested in the West. For example, Merleau-Ponty termed the lived body as the “body in human experience” or the “perceived body” and goes on to explain that this refers to the projection of the body into the real world [28]. Perception is the starting point of consciousness and begins in the body before it is projected to other objects. The somatization of emotional symptoms is not unique in East Asian countries and can be identified across a broad range of cultural backgrounds [19]. Efforts to understand the close and complex relationship between the mind and body are not limited to philosophy and anthropology. Recent studies in the field of neuroscience have suggested that there are mutual interactions between bodily responses and emotions in which physical functions trigger emotional experiences and emotional experiences lead to particular spatial patterns of sensation throughout the body [4, 8, 9].

As Kleinman explained, medical approaches involve “the notions about an episode of sickness and its treatment that are employed by all those engaged in the clinical process” [15, 29]. During this process, notions about illness and treatment are derived from the medical understanding of a particular culture, and East Asian medicine has traditionally employed a unique understanding of the body through its cultural and social conditions. Furthermore, it is interesting to note that the concept of emotions manifesting through the body, as elaborated in the classical medical texts of East Asian medicine and observed in the present study, has evolved over time and now exhibits an understanding that incorporates the influence of Western medicine. Additionally, medical perspectives that understand emotions as an aspect of bodily function or dysfunction within visceral systems have triggered further questions from the perspective of Western medicine in East Asia. The modern articulation of emotional symptoms in biomedical disease categories, such as depression, is an example of the response to the embodiment of an illness. Previous study pointed out that the historical and modern views of East Asian medicine regarding the relationship between emotional disorders and the body indicate that this relationship is man-made and not an objectively defined fact [30]. Emotional diseases that are related to bodily organ systems, such as “Hwa-byung” in Korea and “Utsu” in Japan, which are thought to be based on the influences of modernized East Asian medicine, are examples of “cultural” emotional disorders that have been newly defined in the modern era.

Attempts to understand emotional symptoms through somatization, or in relation to bodily organs, in East Asian medicine have resulted in the classification of emotional diseases within frameworks that are similar to those of Western medicine, even though the direct association of emotional symptoms with bodily organs has been deemed “cultural.” However, attempts to understand such disorders in the modern East using Western medical terms have also led to diagnoses of neurasthenia and depression and the conceptualization of these disorders as “cultural” disorders has only recently been suggested; in fact, the uniquely Korean attributes of Hwa-byung have never been established by a clear consensus [12, 13]. Similarly, the interpretation of emotion-related disorders as predominantly organ system disorders began in modern Japan during the Republican Era, which led to the definition of Utsu as “cultural” disorder [13]. Previous studies have argued that the issue of translation in cultural medical history has not only brought about a linguistic expansion but also led to the conceptual fusion of new ideas [13]. For example, the defining of both Hwa-byung and Utsu raised questions about what was specifically cultural to disorders and in what ways they related to biomedical disease categories and traditional disease concepts of emotional disorders [30].

Western medicine strives to understand humans based on a dichotomy of the psyche and the soma and, as a result, trying to understand emotional symptoms using East Asian practices based on the interpretations and classifications of Western medicine limits the manifestation of these symptoms to being described as “cultural” disorders. When translating “the notions about an episode of sickness and its treatment that are employed by all those engaged in the clinical process,” a comprehensive definition of the sickness and its symptoms is required, within its medical understanding [29]. By focusing on the manifestation of the mind in the body using the principles of East Asian medicine, the present study was an attempt to translate the understanding of emotion as one aspect of bodily functions. A careful translation and interpretation of the knowledge and theories are necessary regarding the systems and functions of the body require attention before the two perspectives are translated into each other's languages. Previous study has shown through a time series analysis that the emotions and physical states are related in a long-term observation even in healthy volunteers, implying for clinical relevance of emotional states and physical states [31]. Furthermore, manipulations of muscular feedback from facial expression can modulate the emotional states, including autonomic responses to the emotional cues [32]. In this regard, a discussion about these two medical viewpoints needs to be established with a comprehensive understanding of the concepts of East Asian medicine in terms of sicknesses and psychosomatic symptoms.

The present study has several limitations. First, the characteristics of emotions as they are related to bodily organ systems were extracted from a single classical Korean medical text with limited bibliographical data. Thus, it will be necessary to further investigate the characteristics of emotion from the perspective of East Asian medicine using other classical medical texts including* Huangdi Neijing*. Second, the available data only allowed for the present data mining method to be applied to the relationship between emotion and the visceral system. Studies on the relationships between emotion and bodily changes, including physical symptoms and *qi* movement, need to be conducted to understand the manifestation of emotion in East Asian medicine and to understand the different approaches to emotion used by Western medicine and East Asian medicine.

In summary, the present study identified specific patterns between emotions and corresponding bodily organ systems. East Asian medicine directly associates an emotion with the visceral system using the language of symptoms rather than that of organs, which can be divided into visible parts. Thus, the different understanding of emotions and their relationship to the body in Western medicine and East Asian medicine have led to distinct interpretations of illness such that Western medicine understands emotional disorders using neural language while East Asian medicine uses somatic language. Understanding these phenomena as well as the actors of medicine can offer a more comprehensive perspective when examining the human body and its symptoms.
